# A blank slate – apropos a clinical case

**DOI:** 10.1192/j.eurpsy.2021.1722

**Published:** 2021-08-13

**Authors:** S. Freitas Ramos, M. Seabra, P.Â. Horta, J. Guimarães, R. Grangeia

**Affiliations:** 1 Department Of Psychiatry And Mental Health, Local Health Unit of Guarda, Guarda, Portugal; 2 Neurology, Faculty of Medicine of the University of Porto, Porto, Portugal; 3 Psychiatry, CHVNG/E, Vila Nova de Gaia, Portugal; 4 Psychiatry, Faculty of Medicine of the University of Porto, Porto, Portugal

**Keywords:** dissociative amnesia

## Abstract

**Introduction:**

Dissociative Amnesia remains an enigmatic and controversial entity. It is classically described as responsible for autobiographic amnesia associated with a traumatic event.

**Objectives:**

To report a clinical case and review the literature.

**Methods:**

We collected data from the patient’s clinical file with his informed consent. We conducted a non-systematic review of the literature.

**Results:**

A 46-years-old patient presents to the emergency department for sudden global retrograde amnesia, with multiple domain amnestic syndrome (impairing verbal and visual memory, processing speed, mental flexibility, calculus, executive functions and language). He was initially admitted for a suspected infectious meningoencephalitis, which was not confirmed. Later an autoimmune encephalitis was pursued. Brain MRI showed a nonspecific left temporal and hipocampal hyperintensity and the EEG a mild left temporal dysfunction. The autoimmune encephalitis panel was negative and the formal diagnostic criteria were not met. The neurologic examination at discharge presented only with autobiographical and semantic amnesia. On the mental state examination, he presented with depressive symptoms reactive to the situation. There was no evident traumatic event apart from a promotion received the day before the amnesia started. He was prescribed escitalopram 10 mg/day. The amnesia was maintained at 9 months follow-up.

**Conclusions:**

Our case report illustrates a case of amnesia without evident organic or psychogenic cause, assumed as a dissociative amnesia. Further studies are necessary to clarify the pathophysiology of this condition and develop specific treatments.

**Disclosure:**

No significant relationships.

